# Production of Vesicular Stomatitis Virus Glycoprotein-Pseudotyped Lentiviral Vector Is Enhanced by Ezrin Silencing

**DOI:** 10.3389/fbioe.2020.00368

**Published:** 2020-04-29

**Authors:** Mai Izumida, Kei Togawa, Hideki Hayashi, Toshifumi Matsuyama, Yoshinao Kubo

**Affiliations:** ^1^Department of Molecular Microbiology and Immunology, Graduate School of Biomedical Sciences, Nagasaki University, Nagasaki, Japan; ^2^Program for Nurturing Global Leaders in Tropical and Emerging Communicable Diseases, Graduate School of Biomedical Sciences, Nagasaki University, Nagasaki, Japan; ^3^Medical University Research Administrator, Nagasaki University School of Medicine, Nagasaki, Japan; ^4^Department of Cancer Stem Cell Biology, Institute of Biomedical Sciences, Nagasaki University, Nagasaki, Japan; ^5^Department of Clinical Medicine, Institute of Tropical Medicine, Nagasaki University, Nagasaki, Japan

**Keywords:** HIV-1, Gag protein, ezrin, VSV-G, phosphorylation

## Abstract

Human immunodeficiency virus type 1 (HIV-1)-based viral vector is widely used as a biomaterial to transfer a gene of interest into target cells in many biological study fields including gene therapy. Vesicular stomatitis virus glycoprotein (VSV-G)-containing HIV-1 vector much more efficiently transduces various mammalian cells than other viral envelope proteins-containing vectors. Understanding the mechanism would contribute to development of a novel method of efficient HIV-1 vector production. HIV-1 vector is generally constructed by transient transfection of human 293T or African green monkey COS7 cells. It was found in this study that HIV-1 Gag protein is constitutively digested in lysosomes of African green monkey cells. Surprisingly, VSV-G elevated HIV-1 Gag protein levels, suggesting that VSV-G protects Gag protein from the lysosomal degradation. Unphosphorylated ezrin, but not phosphorylated ezrin, was detected in COS7 cells, and ezrin silencing elevated Gag protein levels in the presence of VSV-G. Expression of unphosphorylated ezrin reduced Gag protein amounts. These results indicate that unphosphorylated ezrin proteins inhibit the VSV-G-mediated stabilization of HIV-1 Gag protein. Trafficking of HIV-1 Gag-associated intracellular vesicles may be controlled by ezrin. Finally, this study found that ezrin silencing yields higher amount of VSV-G-pseudotyped HIV-1 vector.

## Introduction

A lentiviral vector based on human immunodeficiency virus type 1 (HIV-1) is widely used as a vector to transfer a gene of interest into target cells in many biological study fields including basic biology and clinical gene therapy. Usually, the HIV-1 vector is constructed by transient transfection of human 293T or African green monkey COS7 cells (Narahara and Yasuda, 2015; Pyeon et al., 2015; Kubo et al., 2016; Yang et al., 2016, 2018; Buttler et al., 2018; Iliopoulou et al., 2018; Liu et al., 2019; Sengupta et al., 2019), because these cells express the simian virus 40 T antigen that induces DNA replication of the SV40 replication origin-containing plasmids in transfected cells.

Host cells usually contain restriction mechanisms to inhibit viral replication (Ghimire et al., 2018). Such host restriction mechanisms prevent production of lentiviral vector. Accessory proteins encoded by HIV-1 induce degradation of the host restriction factors to escape from host innate immunity. However, lentivirus vector generally do not express the accessory proteins to prevent unexpected effects of those viral proteins. There is no method for inactivation of host restriction factors to produce high amount of HIV-1 vector particles.

Vesicular stomatitis virus glycoprotein (VSV-G)-containing HIV-1 vector is much more frequently utilized than other viral envelope proteins-containing HIV-1 vectors. When VSV-G-bearing HIV-1 vector is constructed by transient transfection, its transduction titers are generally much higher than those of HIV-1 vectors containing other viral glycoproteins (Cronin et al., 2005). The reason why transduction titer of VSV-G vector is higher than that of other viral glycoprotein-containing vector is unknown. VSV-G may counteract host restriction factors. Understanding the molecular mechanism would contribute to efficient production of lentiviral vector.

Since HIV-1 Gag protein is the major component of viral particles and it is essential for HIV-1 replication, regulation of HIV-1 Gag protein stability is one of the key factors for HIV-1 vector production. There are many lines of evidence showing that HIV-1 Gag protein is degraded in lysosomes. A lysosome inhibitor enhances HIV-1 virion production, suggesting that HIV-1 Gag protein is constitutively degraded in lysosomes (Molle et al., 2009). Rhesus monkey TRIM5α not only inhibits HIV-1 infection (Stremlau et al., 2004), but also induces Gag protein degradation to decrease the number of released viral particles in 293T cells (Sakuma et al., 2007, 2010; Sukegawa et al., 2014). SOCS1 stabilizes Gag protein in 293T cells and silencing of SOCS1 expression by siRNA induces lysosomal degradation of HIV-1 Gag protein (Ryo et al., 2008; Nishi et al., 2009). Apolipoprotein L1 whose expression is induced by interferons restricts HIV-1 replication by inducing lysosomal degradation of the HIV-1 Gag protein (Taylor et al., 2014). Insulin-induced gene 1 inhibits HIV-1 production by lysosomal degradation of Gag protein (Zheng et al., 2019). In this context, VSV-G may affect HIV-1 Gag protein stability. To assess the speculation, in this study we analyzed the impact of VSV-G on the HIV-1 Gag protein levels. We found that VSV-G elevated HIV-1 Gag protein amount in African green monkey cells.

Previously, we have reported that expression of ezrin in target cells is required for CXCR4-tropic HIV-1 infection (Kubo et al., 2008), and expression of ezrin in HIV-1-producing cells is required for infectivity of released HIV-1 particles (Kamiyama et al., 2018). The biological function of ezrin is regulated by its phosphorylation. N- and C-terminal domains of phosphorylated ezrin bind to membrane proteins and actin cytoskeleton, respectively. Trafficking of the membrane proteins is regulated by actin cytoskeleton through phosphorylated ezrin (Pore and Gupta, 2015). N-terminal domain peptide of ezrin (EZ-N) binds to membrane proteins but not actin cytoskeleton. In EZ-N-expressing cells, trafficking of EZ-N-bound membrane proteins is not regulated by actin cytoskeleton. Thus, EZ-N functions as a dominant negative mutant for phosphorylated ezrin. However, N- and C-terminal domains of unphosphorylated ezrin intramolecularly or intermoleculaly interact with each other, and do not bind to membrane protein and actin, respectively.

During our previous study, we noticed that the EZ-N expression in VSV-G-pseudotyped HIV-1 vector-producing cells significantly decreases its transduction titers. Thus, we analyzed the impact of ezrin on VSV-G-mediated stabilization of HIV-1 Gag protein in this study.

## Results

### VSV-G Elevates HIV-1 Gag Protein Amount

**FIGURE 1:**
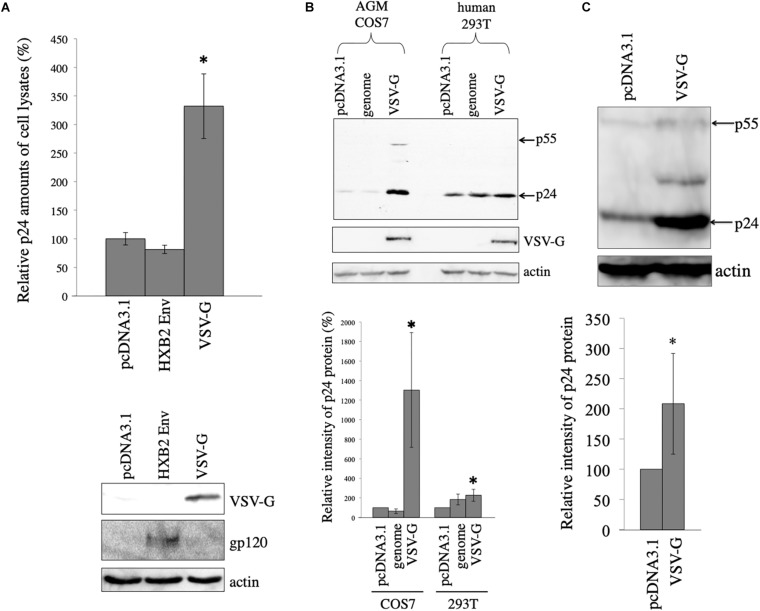
VSV-G elevates HIV-1 Gag protein amount. **(A)** COS7 cells were transfected with the HIV-1 Gag-Pol expression plasmid together with pcDNA3.1, HIV-1 HXB2 Env, or VSV-G expression plasmid. HIV-1 p24 protein amounts in cell lysates were measured by ELISA. Relative values to the p24 amount in pcDNA3.1-transfected cells are indicated (upper panel). Asterisks show significant differences compared to pcDNA3.1. This experiment was repeated three times. Cell lysates from the transfected cells were analyzed by western immunoblotting using indicated antibodies (lower panel). **(B)** AGM COS7 and human 293T cells were transfected with the HIV-1 Gag-Pol expression plasmid together with pcDNA3.1, LacZ-encoding HIV-1 vector genome (genome), or VSV-G expression plasmid. Cell lysates from the transfected cells were analyzed by western immunoblotting (upper panel). HIV-1 Gag precursor (p55) and mature capsid (p24) were indicated by arrows. Intensities of HIV-1 p24 protein bands were measured by a densitometer. Relative values (%) to the intensities in the pcDNA3.1-transfected cells are shown (lower panel). This experiment was repeated three times. Asterisks show significant differences compared to pcDNA3.1. **(C)** African green monkey Vero cells were transfected with HIV-1 Gag-Pol expression plasmid together with pcDNA3.1 or VSV-G expression plasmid. Cell lysates prepared from the transfected cells were analyzed by western blotting (upper panel). Intensities of p24 bands were measured by a densitometer. Relative values to the intensities in the pcDNA3.1-transfected cells are shown (lower panel). This experiment was repeated three times. Asterisks show significant differences compared to pcDNA3.1.

To assess whether viral envelope glycoproteins stabilize HIV-1 Gag protein, African green monkey COS7 cells were transfected with the HIV-1 Gag-Pol expression plasmid lacking the packaging signal together with pcDNA3.1, HXB2 HIV-1 Env, or VSV-G expression plasmid. Cell lysates were prepared from the transfected cells 2 days after the transfection. Gag protein amounts in the cell lysates were measured by p24 ELISA. Gag protein level in the HXB2 Env-expressing cells was similar to that in the pcDNA3.1-transfected cells. However, Gag protein amounts in the VSV-G-expressing cells were three-times higher than those in the pcDNA3.1-transfected cells (Figure 1A). This result indicates that VSV-G elevates HIV-1 Gag protein amount, but HIV-1 Env protein does not.

To assess whether VSV-G also stabilizes HIV-1 Gag protein in human cells, human 293T or African green monkey COS7 cells were transfected with the HIV-1 Gag-Pol expression plasmid together with VSV-G expression plasmid. As controls, 293T or COS7 cells were transfected with the HIV-1 Gag-Pol expression plasmid together with pcDNA3.1 or LacZ-encoding HIV-1 vector genome expression plasmid (genome). Cell lysates were prepared from the transfected cells 2 days after the transfection, and were analyzed by western immunoblotting. HIV-1 Gag p24 protein band intensity was elevated 19 and 2 times by VSV-G expression compared to that in pcDNA3.1 in COS7 and 293T cells, respectively (Figure 1B). The pcDNA3.1 or LacZ-encoding HIV-1 vector genome expressing plasmid did not elevate HIV-1 Gag protein amounts. Level of p24 was very low in the absence of VSV-G (10-s exposure time). However, when the blot was exposed longer time (3 min), p24 protein bands were easily detected. These results show that VSV-G elevates HIV-1 Gag protein amount especially in African green monkey COS7 cells.

To examine whether VSV-G elevates HIV-1 Gag protein amount in other African green monkey cells, Vero cells were transfected with the HIV-1 Gag-Pol expression plasmid together with pcDNA3.1 or VSV-G expression plasmid. Cell lysates were prepared from the transfected cells 2 days after the transfection. Gag protein expression levels in the transfected cells were analyzed by western immunoblotting using anti-p24 antibody. VSV-G increased HIV-1 Gag protein amounts 2 times in African green monkey Vero cells (Figure 1C). These results suggests that VSV-G elevates HIV-1 Gag protein in all three cell lines used in this experiment.

### Elevation of Gag Protein Level by VSV-G Is Not Induced by Retro-Transduction

**FIGURE 2:**
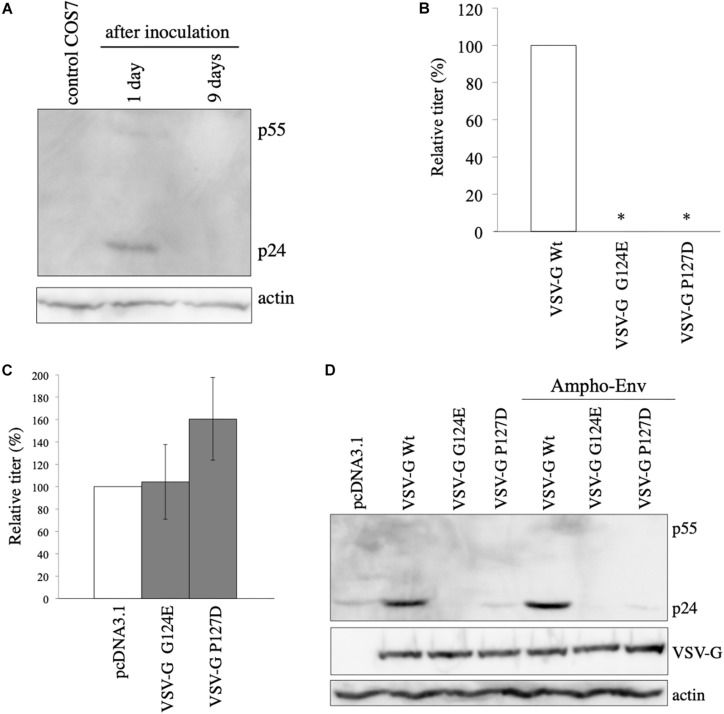
VSV-G-mediated increase of HIV-1 Gag protein is not induced by retro-transduction. **(A)** COS7 cells were inoculated with VSV-G-pseudotyped HIV-1 vector. Cell lysates were prepared from the inoculated cells 1 and 9 days after the inoculation. Gag protein was analyzed by western blotting. HIV-1 Gag precursor (p55) and mature capsid (p24) were indicated. **(B)** Transduction titers of HIV-1 vector carrying VSV-G Wt, G124E, or P127D were measured. Relative values to the transduction titers of VSV-G Wt-carrying HIV-1 vector are indicated (*n* = 3). Asterisks show significant differences compared to VSV-G Wt. **(C)** 293T cells were transfected with amphotropic MLV-pseudotyped HIV-1 vector construction plasmids together with VSV-G G124E- or P127D plasmid. Relative values to the transduction titers of pcDNA3.1-transfected cells are shown (*n* = 3). **(D)** 293T cells were transfected with HIV-1 Gag-Pol expression plasmid together with VSV-G Wt, G124E, or P127D plamid in the absence or presence of amphotropic MLV Env expression plasmid. Cell lysates were prepared from the transfected cells. Gag, VSV-G, and actin proteins were analyzed by western blotting.

It has been previously reported that VSV-G elevates HIV-1 Gag protein by retro-transduction in the replication-defective VSV-G-pseudotyped HIV-1, when a packaging signal-containing HIV-1 Gag-Pol expression plasmid is used (Ohishi et al., 2007). However, in our study, African green monkey cells that are resistant for HIV-1 infection (Keckesova et al., 2004) were used. Indeed, when VSV-G-pseudotyped HIV-1 vector was inoculated to COS7 cells, LacZ marker-expressing cells were not detected (unpublished observation). Furthermore, the HIV-1 Gag-Pol expression plasmid used in this study lacks the packaging signal (Naldini et al., 1996). To know whether HIV-1 *gag* gene is transmitted to inoculated COS7 cells, Gag protein expression in COS7 cells inoculated with undiluted VSV-G-pseudotyped HIV-1 vector was analyzed by western blotting 1 and 9 days after the inoculation. Gag p24 protein was not detected 9 days after the inoculation, indicating that HIV-1 *gag* gene is not transmitted to the inoculated COS7 cells (Figure 2A). Gag protein was slightly detected 1 day after the inoculation, suggesting that this signal corresponds to Gag protein bound to COS7 cell surface that is detached or degraded during several passages. These results show that the VSV-G-mediated increase of Gag protein level is not induced by retro-transduction.

### VSV-G Membrane Fusion Activity Is Required for Its Ability to Elevate Gag Protein Level

To further confirm the conclusion that the VSV-G-mediated elevation of Gag protein is not induced by retro-transduction, we used VSV-G mutants (G124E and P127D) deficient for fusion activity (Ohishi et al., 2007). To confirm whether the VSV-G mutants do not induce vector infection, COS7 cells were transfected with HIV-1 Gag-Pol and LacZ-encoding HIV-1 vector genome expression plasmids together with VSV-G Wt, G124E, or P127D expression plasmid. Culture supernatants were collected from the transfected cells 2 days after the transfection, and were inoculated to TE671 cells. The inoculated cells were stained with X-Gal 2 days after the inoculation, and numbers of blue cells were counted. Transduction titers of the VSV-G mutant-pseudotyped HIV-1 vector were much lower than that those of the Wt VSV-G-containing vector (Figure 2B), as expected.

To assess whether the VSV-G mutants enhance HIV-1 Gag protein amount, COS7 cells were transfected with amphotropic MLV-pseudotyped HIV-1 vector construction plasmids together with pcDNA3.1, G124E, or P127D mutant expression plasmid, and cell lysates were prepared from the transfected cells 2 days after the transfection. Transduction titers were not elevated by the G124E VSV-G (Figure 2C). The P127D mutant slightly elevated transduction titers, but the difference was not statistically significant. Consistently, HIV-1 Gag protein levels were not increased by the VSV-G mutants (Figure 2D). These results suggest that the VSV-G-mediated stabilization of HIV-1 Gag protein requires the membrane fusion activity of VSV-G protein.

### HIV-1 Gag Protein Is Digested in Lysosomes

**FIGURE 3:**
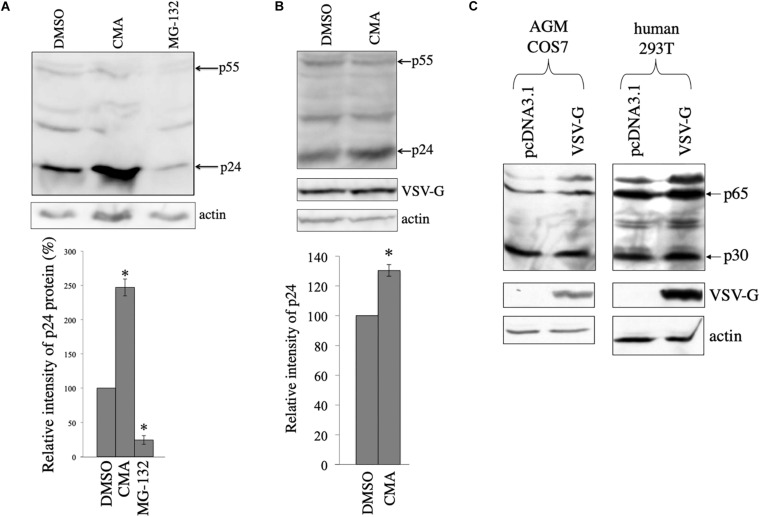
HIV-1 Gag protein is digested in lysosomes and VSV-G rescues Gag protein from the digestion in COS7 cells. **(A)** COS7 cells were transfected with the HIV-1 Gag-Pol expression plasmid, and then were treated with DMSO, CMA, or MG-132. Cell lysates from the transfected cells were analyzed by western immunoblotting (upper panel). HIV-1 Gag precursor (p55) and mature capsid (p24) were indicated by arrows. Intensities of the p24 protein bands were measured by a densitometer. Relative values to the intensities in the pcDNA3.1-transfected cells are indicated (lower panel, *n* = 3). Asterisks show significant differences compared to DMSO. **(B)** COS7 cells were transfected with the HIV-1 Gag-Pol and VSV-G expression plasmids, and were treated with DMSO or CMA. Cell lysates prepared from the treated cells were analyzed by western blotting. Intensities of the p24 protein bands were measured by a densitometer. Relative values to the intensities in the pcDNA3.1-transfected cells are indicated (lower panel, *n* = 3). Asterisks show significant differences compared to DMSO. **(C)** AGM COS7 and human 293T cells were transfected with the MLV Gag-Pol expression plasmid together with pcDNA3.1 or VSV-G expression plasmid. Cell lysates prepared from the transfected cells were analyzed by western blotting. MLV Gag precursor (p65) and mature capsid (p30) were indicated by arrows.

In other words, the above results suggest that HIV-1 Gag protein is unstable in the absence of VSV-G. To assess whether HIV-1 Gag protein is digested in lysosomes or in proteasomes, COS7 cells were transfected with the HIV-1 Gag-Pol expression plasmid, and then were treated with a lysosome inhibitor, concanamycin A (CMA) (3 nM), or a proteasome inhibitor, MG-132 (10 μM) for 5 h, 1 day after the transfection. The CMA treatment elevated HIV-1 Gag protein levels 2.5 times, but the MG-132 treatment did not (Figure 3A). This result shows that HIV-1 Gag protein is constitutively degraded in lysosomes, as already reported (Molle et al., 2009).

To assess whether HIV-1 Gag protein is degraded in the presence of VSV-G, COS7 cells were transfected with HIV-1 Gag-Pol expression plasmid together with VSV-G expression plasmid, and cultured for 24 h. The transfected cells were treated with DMSO or CMA for 5 h. Cell lysates were prepared from the transfected cells 24 h after the treatment, and western blotting was performed. Gag amounts were elevated 1.3 times by the CMA treatment in the presence of VSV-G (Figure 3B), but its efficiency was lower than that in the absence of VSV-G (Figure 3A). This result confirm the above conclusion that VSV-G protects HIV-1 Gag protein from lysosomal degradation in COS7 cells.

To examine whether VSV-G elevates murine leukemia virus (MLV) Gag protein levels, COS7 and 293T cells were transfected with the MLV Gag-Pol expression plasmid together with pcDNA3.1 or VSV-G expression plasmid. Cell lysates were prepared from the transfected cells 2 days after the transfection. MLV Gag protein amounts were not altered by VSV-G (Figure 3C). This result shows that VSV-G stabilizes HIV-1 Gag protein, but not MLV Gag protein.

### Ezrin Is Involved in VSV-G-Mediated Stabilization of HIV-1 Gag Protein

We have previously reported that ezrin expressed in target cells is required for efficient CXCR4-tropic HIV-1 infection (Kubo et al., 2008). Since ezrin protein is incorporated into HIV-1 particles (Kamiyama et al., 2018), ezrin expressed in HIV-1-producing cells might affect HIV-1 virion production and/or infectivity of released particles. We have previously reported that ezrin expressed in HIV-1 Env-containing HIV-1 vector-producing cells is important for infectivity of released vector particles, but not for virion production (Kamiyama et al., 2018). During the study, we noticed that an ezrin dominant negative mutant (EZ-N) (Algrain et al., 1993) significantly reduced Gag protein levels in cell lysates, when VSV-pseudotyped HIV-1 vector was used.

**FIGURE 4:**
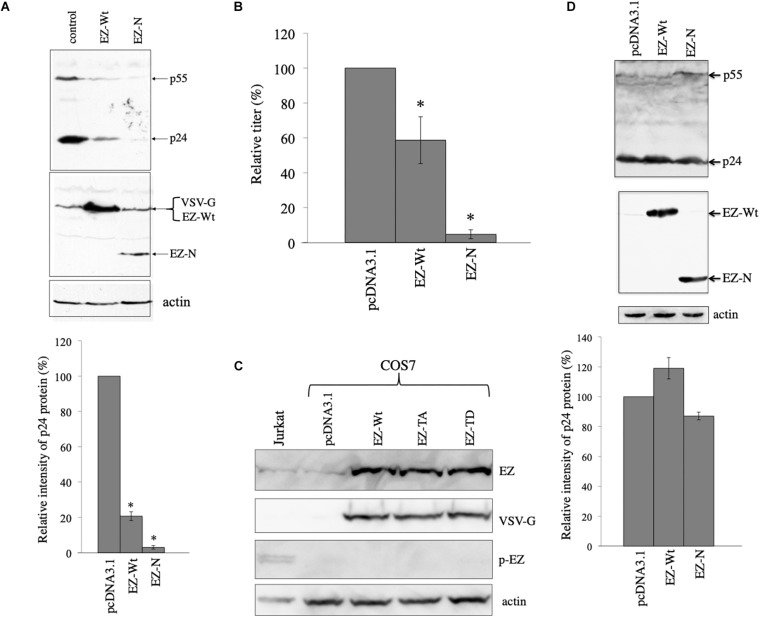
Ezrin is involved in the VSV-G-mediated stabilization of HIV-1 Gag protein. **(A)** COS7 cells were transfected with the VSV-G-pseudotyped HIV-1 vector construction plasmids together with pcDNA3.1 (control), VSV-G-tagged ezrin wild type (EZ-Wt), or VSV-G-tagged ezrin dominant negative mutant (EZ-N) expression plasmid. Cell lysates from the transfected cells were analyzed by western immunoblotting (upper panel). HIV-1 Gag precursor (p55), mature capsid (p24), EZ-Wt, EZ-N, and VSV-G proteins were indicated by arrows. Ez-Wt and VSV-G were not separated. Intensities of the HIV-1 p24 protein bands were measured by a densitometer. Relative values to the intensities in the pcDNA3.1-transfected cells are indicated (lower panel, *n* = 3). Asterisks show significant differences compared to pcDNA3.1. **(B)** Culture supernatants from the transfected cells were inoculated into HeLa cells, and transduction titers were counted. Transduction titers in the pcDNA3.1-transfected cells were always set to 100. Relative values to the titers in the pcDNA3.1-transfected cells are indicated. This experiment was repeated three times, and means ± standard deviations are indicated. Asterisks show significant differences compared to pcDNA3.1. **(C)** COS7 cells were transfected with pcDNA3.1, EZ-Wt, EZ-TA, or EZ-TD expression plasmid. Cell lysates from the transfected COS7 and Jurkat cells were analyzed by western blotting using the indicated antibodies. **(D)** COS7 cells were transfected with the HXB2 Env-containing HIV-1 vector construction plasmids together with pcDNA3.1, EZ-Wt, or EZ-N expression plasmid. Cell lysates from the transfected cells were analyzed by western immunoblotting (upper panel). Intensities of p24 protein bands were measured. Relative values to the intensities in the pcDNA3.1-transfected cells are shown (lower panel, *n* = 3).

To confirm the result, COS7 cells were transfected with the VSV-pseudotyped HIV-1 vector construction plasmids together with pcDNA3.1, VSV-G-tagged human ezrin wild type (EZ-Wt), or VSV-G-tagged EZ-N expresion plasmid (Algrain et al., 1993). Since only 5 residues are changed among 586 amino acids between human and green monkey ezrin sequences (Supplementary Figure 1), human EZ-Wt and EZ-N should function in COS7 cells as in human cells. Cell lysates were prepared from the transfected cells 2 days after the transfection, and were analyzed by western blotting. EZ-Wt and EZ-N reduced HIV-1 Gag protein amounts to 20% and 2% of those in pcDNA3.1-transfected cells, respectively (Figure 4A). Since molecular sizes of the EZ-Wt and VSV-G proteins were same, these protein bands were not separated. Transduction titers of culture supernatants from the transfected cells were also decreased by the EZ-Wt and EZ-N (Figure 4B). These results suggest that ezrin reduces HIV-1 Gag protein amount in the presence of VSV-G.

Biological function of ezrin is controlled by its phosphorylation as described in the Section “Introduction”. We analyzed whether phosphorylated ezrin is detected in COS7 cells by western blotting. Total ezrin protein was detected, but phosphorylated ezrin was not in COS7 cells (Figure 4C). Phosphorylated ezrin (p-EZ) was detected in Jurkat T cells, as previously reported (Kamiyama et al., 2018). This result indicates that ezrin protein is expressed, but is not phosphorylated in COS7 cells.

To examine whether the ezrin proteins reduce HIV-1 Gag protein in the absence of VSV-G, COS7 cells were transfected with the CXCR4-tropic Env-containing HIV-1 vector construction plasmids together with pcDNA3.1, EZ-Wt, or EZ-N expression plasmid. Cell lysates were prepared from the transfected cells 2 days after the transfection. The EZ-Wt and EZ-N did not affect HIV-1 Gag protein levels (Figure 4D). These results indicates that ezrin is involved in the VSV-G-mediated stabilization of HIV-1 Gag protein.

**FIGURE 5:**
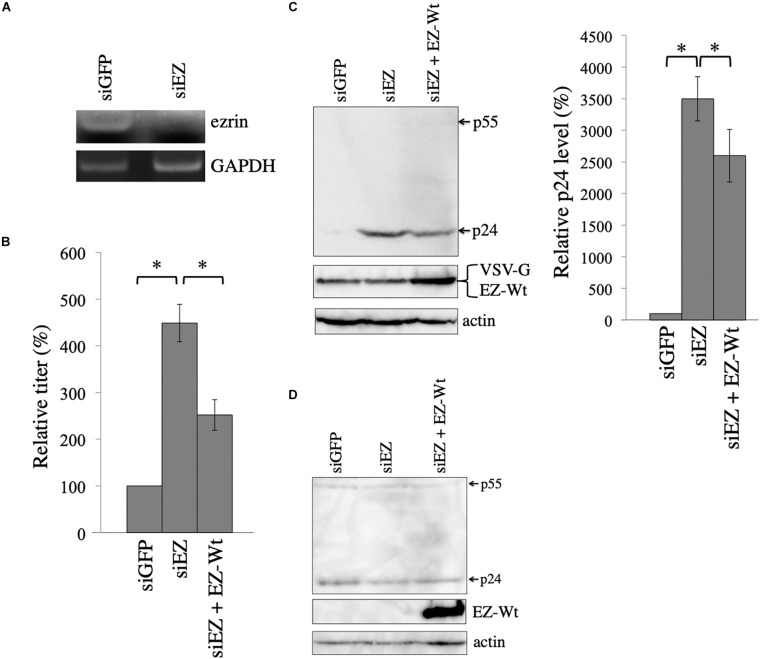
Ezrin silencing elevates HIV-1 Gag protein level. **(A)** COS7 cells were transfected with the VSV-pseudotyped HIV-1 vector construction plasmids together with siGFP or siEZ. Total RNA samples were isolated from the transfected cells. GAPDH and ezrin mRNA levels were analyzed by semi-quantitative RT-PCR. **(B)** Culture supernatants from the transfected cells were inoculated to target cells, and transduction titers were measured. Relative values to the transduction titers in siGFP-transfected cells are indicated (*n* = 3). Asterisks show significant differences between the indicated groups. **(C)** Cell lysates prepared from the transfected cells were analyzed by western blotting. HIV-1 Gag precursor (p55), mature capsid (p24), and EZ-Wt proteins were indicated (left panel). Intensities of p24 protein were measured by a densitometer. Relative values to the intensities in the siGFP-transfected cells are shown (right panel, *n* = 3). Asterisks show significant differences between the indicated groups. **(D)** COS7 cells were transfected with HIV-1 HXB2 Env-containing HIV-1 vector construction plasmids together with siGFP or siEZ. Cell lysates from the transfected cells were analyzed with western blotting.

To confirm that VSV-G-mediated stabilization of HIV-1 Gag protein is dependent on ezrin, COS7 cells were transfected with the VSV-G-pseudotyped HIV-1 vector construction plasmids together with siRNA against GFP (siGFP) or ezrin (siEZ). Total RNA samples were prepared from the transfected cells 2 days after the transfection. Ezrin mRNA levels of the siEZ-transfected cells were lower than those of the siGFP-transfected cells analyzed by RT-PCR, confirming ezrin silencing (Figure 5A). Anti-ezrin antibody additionally detects radixin, another member of ezrin-radixin-moesin (ERM) family, and these molecular sizes were same (Kamiyama et al., 2018). Thus, RT-PCR was performed to examine the ezrin silencing.

Culture supernatants were collected from the transfected cells 2 days after the transfection, and were inoculated to 293T cells. The siEZ transfection significantly increased transduction titers 4.5 times (Figure 5B). When ezrin expression was recovered by co-transfection with the EZ-Wt expression plasmid, transduction titers were decreased. The siEZ targets the 3’ untranslated region of ezrin mRNA, and the EZ-Wt expression plasmid does not contain the untranslated region (Kubo et al., 2008). Thus, the siEZ should have no effect on the EZ-Wt expression. Western blot analysis of the transfected cells reveals that HIV-1 Gag protein levels were increased by siEZ (Figure 5C). Corresponding to the transduction titers, the EZ-Wt expression reduced HIV-1 Gag protein amounts. When COS7 cells were transfected with the HIV-1 Gag-Pol expression plasmid together with siGFP or siEZ in the absence of VSV-G expression plasmid, HIV-1 Gag protein amount was not changed (Figure 5D). These results suggest that endogenous ezrin inhibits the VSV-G-mediated stabilization of HIV-1 Gag protein, and ezrin silencing significantly enhances production of VSV-G-pseudotyped HIV-1 vector.

**FIGURE 6:**
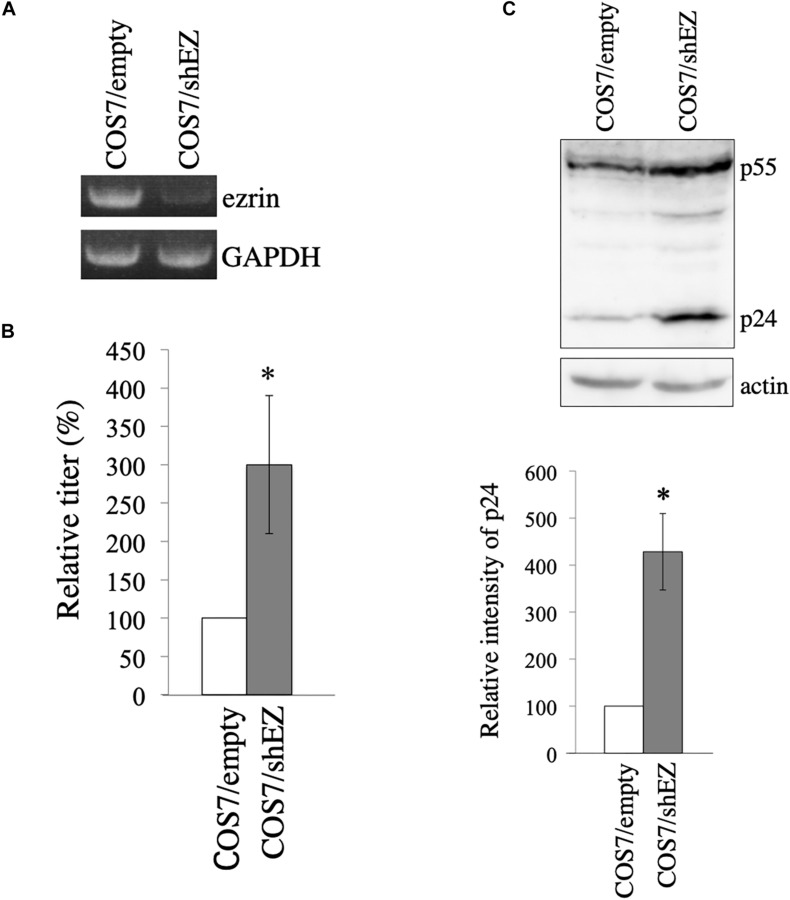
Construction of COS7 cells stably silenced ezrin. **(A)** Levels of ezrin and GAPDH mRNAs in COS7/empty and COS7/shEZ cells were analyzed by RT-PCR. **(B)** COS7/empty and COS7/shEZ cells were transfected with VSV-G-pseudotyped HIV-1 vector construction plasmids. Culture supernatants from the transfected cells were inoculated to TE671 cells, and transduction titers were measured. Relative values to the transduction titers in the COS7/empty cells are indicated (*n* = 3). Asterisks show significant differences compared to COS7/empty cells. **(C)** Cell lysates prepared from the transfected cells were analyzed by western blotting using p24 and actin antibodies. HIV-1 Gag precursor (p55) and mature capsid (p24) were indicated (upper panel). Intensities of p24 protein were measured by a densitometer. Relative values to the intensities in the COS7/empty cells are shown (lower panel, *n* = 3). Asterisks show significant differences compared to COS7/empty cells.

To construct COS7 cells in which ezrin expression is stably silenced, a VSV-pseudotyped MLV vector encoding shRNA against ezrin (shEZ) was inoculated to COS7 cells, and the inoculated cells were selected with G418, because the MLV vector additionally expresses the neomycin resistant gene. Target sequence of the shEZ was same as that of the siEZ used in the above experiment. The G418-resistant cell pool (COS7/shEZ) was used in the following experiment. Level of ezrin mRNA in the COS7/shEZ cells was indeed lower than that in control COS7 cells, analyzed by RT-PCR (Figure 6A). Control and COS7/shEZ cells were transfected with the VSV-G-pseudotyped HIV-1 vector construction plasmids, and culture supernatants from the transfected cells were inoculated to TE671 cells to measure transduction titers. Transduction titers from the transfected COS7/shEZ cells were higher than those from the control cells (Figure 6B). Consistently, HIV-1 p24 amounts were elevated by the shEZ expression (Figure 6C). These results strongly support the above conclusion that endogenous ezrin inhibits HIV-1 Gag expression and HIV-1 vector production in the presence of VSV-G.

### Role of Ezrin Phosphorylation on VSV-G-Mediated Stabilization of HIV-1 Gag Protein

**FIGURE 7:**
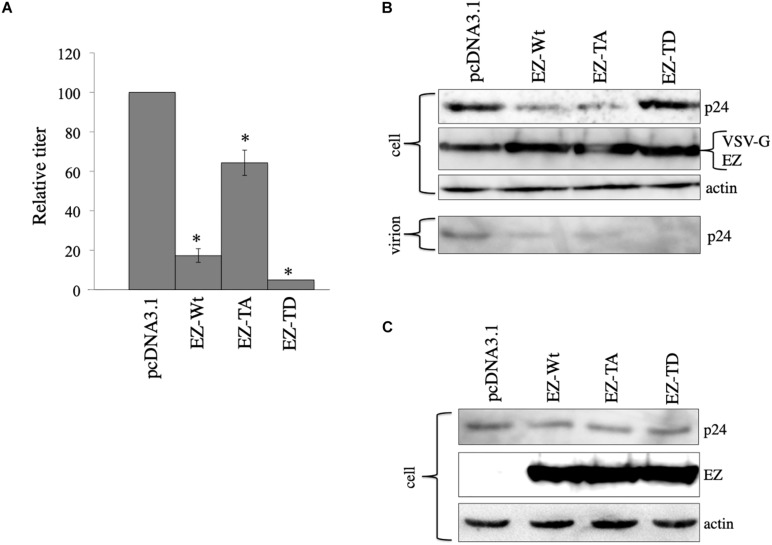
Role of ezrin phosphorylation on VSV-G-mediated stabilization. **(A)** COS7 cells were transfected with the VSV-pseudotyped HIV-1 vector construction plasmids together with pcDNA3,1, EZ-Wt, EZ-TA, or EZ-TD expression plasmid. Culture supernatants from the transfected cells were inoculated to HeLa cells, and transduction titers were measured. Relative values to the transduction titers in the pcDNA3.1-transfected cells are indicated (*n* = 3). Asterisks show significant differences compared to pcDNA3.1. **(B)** Cell lysates prepared from the transfected cells were analyzed by western blotting. HIV-1 mature capsid (p24), EZ, and VSV-G proteins were indicated. **(C)** COS7 cells were transfected with the expression plasmids for construction of the HIV-1 vector without any viral Env protein together with pcDNA3.1, EZ-Wt, EZ-TA, or EZ-TD expression plasmid. Cell lysates from the transfected cells were analyzed by western blotting.

We constructed ezrin mutants that contain amino acid substitutions at threonine residue to alanin (EZ-TA) and aspartic acid (EZ-TD) (Kamiyama et al., 2018). EZ-TA cannot be phosphorylated, and EZ-TD functions as phosphorylated ezrin. To know the effects of these ezrin mutants on the VSV-G-mediated stabilization of HIV-1 Gag protein, COS7 cells were transfected with the VSV-G-pseudotyped HIV-1 vector construction plasmids together with pcDNA3.1, EZ-Wt, EZ-TA, or EZ-TD expression plasmid. Cell lysates were prepared from the transfected cells 2 days after the transfection. Expression of these ezrin mutants did not induce ezrin phosphorylation (Figure 4C). Culture supernatants were collected from the transfected cells 2 days after the transfection, and transduction titers were measured. EZ-Wt attenuated transduction titers to about 20% compared to transduction titers of control VSV-pseudotyped HIV-1 vector (Figure 7A) like the above result (Figure 4B). EZ-TA and -TD mutants decreased to 50% and 5% of the control transduction titers, respectively.

To examine effects of the ezrin mutants on Gag protein levels, cell lysates, and virion fractions prepared from the transfected cells were analyzed by western immunoblotting. Consistent with the transduction titers, EZ-Wt and EZ-TA reduced Gag protein levels in cell lysates (Figure 7B). Gag protein amounts in the EZ-TD-transfected cells were comparable to those in control cells, but those in the virion fraction were significantly reduced by EZ-TD. These results suggest that unphosphorylated ezrin decreases Gag levels in HIV-1 vector-producing cells, and phosphorylated ezrin inhibits HIV-1 virion production, as we already reported (Kamiyama et al., 2018). In the absence of VSV-G, EZ-Wt, EZ-TA, and EZ-TD did not affect HIV-1 Gag protein levels in cell lysates (Figure 7C). This result also supports the above conclusion that ezrin is associated with the VSV-G-mediated stabilization of HIV-1 Gag protein.

### Cellular Localization of VSV-G, EZ-TA, and EZ-TD Proteins

**FIGURE 8:**
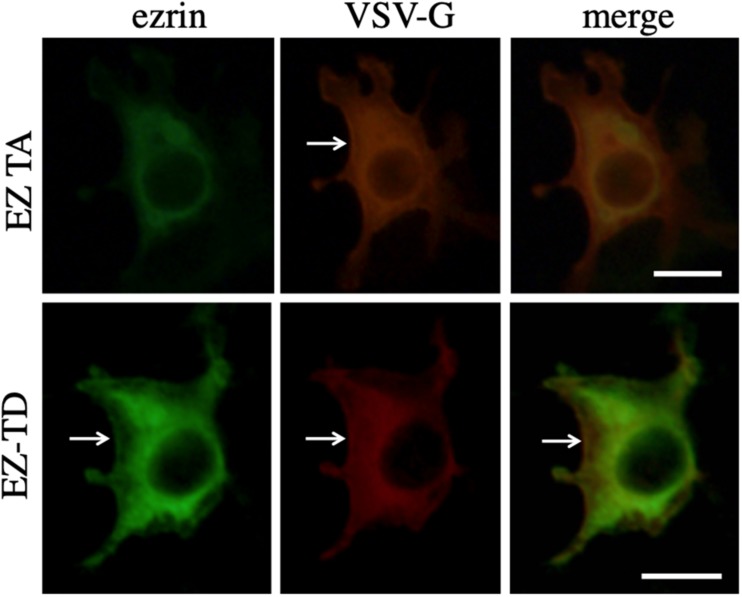
Cellular localization of VSV-G with EZ-TA or EZ-TD. COS7 cells were transfected with the VSV-G expression plasmid together with the EZ-TA or EZ-TD expression plasmid. The cells were stained with mouse anti-VSV-G and goat anti-ezrin antibodies, and then Cy3-conjugated anti-mouse IgG (red) and FITC-conjugated-anti-goat IgG (green) antibodies. Scale bar is 10 μm. White arrows indicate cell surface.

To investigate the interaction between VSV-G and ezrin protein, COS7 cells were transfected with the VSV-G expression plasmid together with the EZ-TA or EZ-TD expression plasmid. The transfected cells were permeabilized with methanol 2 days after the transfection, and treated with mouse anti-VSV-G and goat anti-ezrin antibodies then with Cy3 (red)-conjugated anti-mouse IgG and FITC (green)-conjugated anti-goat IgG antibodies. These proteins were distributed to cytoplasm. EZ-TD and VSV-G proteins were additionally detected in cell surface membrane (Figure 8, arrows). Cellular localization of VSV-G protein was not changed in EZ-TA- or EZ-TD-transfected cells. This result suggests that ezrin indirectly controls the VSV-G-mediated HIV-1 Gag transport.

## Discussion

Vesicular stomatitis virus glycoprotein-pseudotyped HIV-1 vector is widely utilized to transfer a gene of interest into target cells. This study found that VSV-G rescues HIV-1 Gag protein from lysosomal degradation, and unphosphorylated ezrin inhibits the VSV-G-mediated stabilization of Gag protein in COS7 cells. Ezrin silencing significantly enhanced HIV-1 vector production in COS7 cells. VSV-G also enhanced Gag protein levels in 293T and Vero cells, thought the efficiency was lower than in COS7 cells. Thus, the strategy to enhance production of HIV-1 vector by ezrin silencing is useful for many study fields using the HIV-1 vector.

It was also found that HIV-1 Gag protein is constitutively degraded in lysosomes, as already reported (Molle et al., 2009). Consistently, there are several lines of evidence showing that HIV-1 Gag protein localizes to late endosomes/lysosomes (Sherer et al., 2003; Grigorov et al., 2006; Perlman and Resh, 2006). Especially in COS7 cells, HIV-1 Gag protein was much more efficiently digested than in 293T and Vero cell lines. The lysosomal degradation of HIV-1 Gag protein reduces HIV-1 virion production, and might be one of the host defense mechanisms against HIV-1. Although it has been reported that rhesus monkey TRIM5α decreased HIV-1 Gag protein amount (Sakuma et al., 2007), the co-transfection of 293T cells with HIV-1 Gag-Pol and African green monkey TRIM5α expression plasmids did not affect the HIV-1 Gag protein levels (unpublished data), suggesting that African green monkey TRIM5α is dispensable for the degradation of Gag protein in COS7 cells.

Interestingly, VSV-G rescued HIV-1 Gag protein from lysosomal degradation, and ezrin silencing enhanced the VSV-G-mediated Gag stabilization. Total ezrin protein, but not phosphorylated ezrin, was detected in COS7 cells (Kamiyama et al., 2018), showing that unphosphorylated ezrin is expressed in COS7 cells. The ezrin silencing significantly increased HIV-1 Gag protein levels. Furthermore, EZ-Wt and EZ-TA decreased HIV-1 Gag protein levels. Taken together, these results suggest that unphosphorylated ezrin inhibits the VSV-G-mediated stabilization of HIV-1 Gag protein. EZ-TA less efficiently decreased transduction titers than EZ-Wt. The amino acid substitution from threonine to alanine may affect the function.

Ezrin that functions as a dominant negative mutant of phosphorylated ezrin significantly inhibited the VSV-G-mediated stabilization of HIV-1 Gag protein, suggesting that phosphorylated ezrin facilitates the stabilization of Gag protein. However, EZ-TD did not elevate Gag protein levels, and phosphorylated ezrin protein was not detected in COS7 cells. Thus, the role of phosphorylated ezrin on the VSV-G-mediated Gag stabilization is not clear. In contrast, EZ-TD significantly decreased transduction titers and Gag p24 amounts in virion fractions. As we have previously reported (Kamiyama et al., 2018), this result indicated that phosphorylated ezrin suppresses HIV-1 particle production.

Vesicular stomatitis virus glycoprotein tagging inhibits the activity of ezrin to decrease HIV-1 Gag amount. In control and ezrin-silenced COS7 cells, EZ-Wt decreased HIV-1 Gag protein level to 20 and 80%, respectively. Level of endogenous ezrin was much lower than that of exogenous EZ-Wt (Kubo et al., 2008). The ezrin silencing elevated Gag amount 35 times, and overexpression of EZ-Wt decreased Gag level to 20%. This result indicates that endogenous ezrin has much higher activity to decrease Gag level than exogenous VSV-G-tagged EZ-Wt. The VSV-G tagging may inhibit the activity.

Taken together, it is thought that following events take place in each cell as follows. (1) In control COS7 cells, endogenous unphosphorylated ezrin protein is expressed. Unphosphorylated ezrin inhibits the VSV-G-mediated HIV-1 Gag stabilization, but its level is relatively lower. Thus, the endogenous unphosphorylated ezrin moderately inhibits VSV-G-mediated Gag stabilization, and VSV-G expression can stabilize Gag protein and enhance transduction titers. (2) In EZ-TA-transfected cells, level of unphosphorylated ezrin protein is increased, and VSV-G-mediated Gag stabilization is suppressed. As the result, transduction titers are attenuated. (3) In EZ-Wt-transfected cells, level of unphosphorylated ezrin protein is increased, as in EZ-TA-transfected cells, because ezrin kinase activity is too low to phosphorylate EZ-Wt protein. Therefore, VSV-G-mediated Gag stabilization is inhibited, and transduction titers are reduced. (4) The EZ-N protein functions as a dominant negative mutant of phosphorylated ezrin. Thus, phosphorylated ezrin may promote the VSV-G-mediated Gag stabilization, because EZ-N transfection decreased Gag protein amount and transduction titers. However, phosphorylated ezrin protein was not detected in COS7 cells. Since the EZ-TD mutant did not affect HIV-1 Gag amount, phosphorylated ezrin is not involved in the VSV-G-mediated stabilization of Gag protein. The EZ-N may affect function of unphosphorylated ezrin protein. (5) In siEZ-transfected cells, the function of unphosphorylated ezrin to suppress the VSV-G-mediated Gag stabilization is inhibited, because unphosphorylated ezrin is expressed in COS7 cells. As the result, Gag levels and transduction titers are elevated. (6) In EZ-TD-transfected cells, Gag protein levels were not elevated. EZ-TD may have no effect on the VSV-G-mediated stabilization of HIV-1 Gag protein. In contrast, EZ-TD attenuated transduction titers by the inhibition of virion production.

**FIGURE 9:**
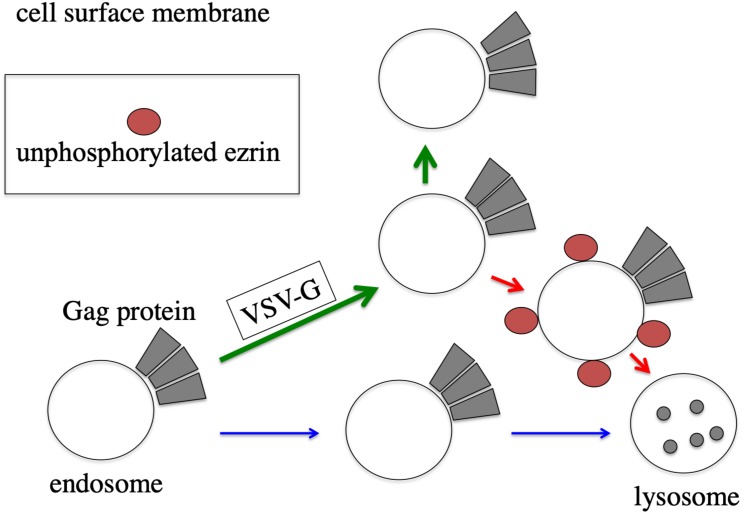
Speculated model of ezrin-dependent stabilization of HIV-1 Gag protein by VSV-G in African green monkey cells. In the absence of VSV-G, HIV-1 Gag protein-associated endosomes are transported to lysosomes, and HIV-1 Gag protein is degraded (blue arrows). In the presence of VSV-G, trafficking of HIV-1 Gag protein-associated endosomes is changed to cell surface (green arrows). Even in the presence of VSV-G, unphosphorylated ezrin induces the transportation of HIV-1 Gag-associated endosomes to lysosomes (red arrows).

Ezrin controls VSV-G-mediated vesicle transport. It has been reported that ezrin functions in endosome sorting (Chirivino et al., 2011; Kvalvaag et al., 2013). Ezrin might control transport of endosomes associated with HIV-1 Gag protein (Figure 9). In the presence of VSV-G, unphosphorylated ezrin transport HIV-1 Gag-associated endosomes to lysosomes. However, VSV-G protein did not form a complex with ezrin protein, analyzed by immunoprecipitation (unpublished). Cellular localization of VSV-G was not changed in EZ-TA- or EZ-TD-expressing cells. These results suggest that VSV-G does not directly interact with ezrin protein. Trafficking of VSV-G protein-containing endosomes may be regulated by ezrin. In the presence of VSV-G, HIV-1 Gag protein may be transported via ezrin-dependent vesicles, and ezrin may affect the fate of the vesicles, but not in the absence of VSV-G. Additionally, this study showed that membrane fusion activity of VSV-G is required for the stability of HIV-1 Gag protein. The membrane fusion activity may regulate transport of intracellular vesicles, but the mechanism is unknown.

Ezrin is required for HIV-1 Env-mediated vector infection. We have previously reported that ezrin silencing attenuates infectivity of released HIV-1 vector particles containing HIV-1 Env protein, suggesting that HIV-1 Env-mediated infection requires ezrin expressed in HIV-1 vector-producing cells (Kamiyama et al., 2018). However, in this study, ezrin silencing rather elevated transduction titers of VSV-pseudotyped HIV-1 vector by increasing HIV-1 Gag protein amounts. Ezrin protein may be required for the HIV-1 Env-meCAGAATAATCGCGAGdiated infection but not VSV-G-mediated infection.

Hosts have defense mechanisms against virus infections. The host defense mechanisms disturb production of HIV-1 vector and inhibit gene transfer by HIV-1 vector. Although the accessary factors encoded by HIV-1 genome counteract some of the host defense mechanisms, HIV-1 vector deficient for the accessary genes is generally used to eliminate unexpected effects induced by these viral proteins. In addition to the accessary genes of HIV-1, some of viral envelope proteins counteract the host defense mechanisms. Envelope proteins (Envs) of HIV-2, simian immunodeficiency virus (SIV), and Ebola virus neutralize BST-2-mediated inhibition of virion release (Gupta et al., 2009; Le Tortorec and Neil, 2009; Hauser et al., 2010; Lopez et al., 2010; Beitari et al., 2019). VSV-G may counteract a host defense mechanism to degrade VSV core protein in lysosomes, and this VSV-G activity may also protect HIV-1 Gag protein from the host defense mechanism.

In summary, this study found that ezrin silencing significantly enhances HIV-1 vector production. In addition, it was suggested that African green monkey cells have a host defense mechanism that digest HIV-1 Gag protein in lysosomes. VSV-G protein counteracts the lysosomal degradation of HIV-1 Gag protein through ezrin-dependent endosome sorting pathway. The VSV-G-mediated stabilization of HIV-1 Gag protein is one of the reasons why transduction titer of VSV-G-pseudotyped HIV-1 vector is generally higher than that of other viral envelope proteins-containing HIV-1 vectors.

## Materials and Methods

### Cells

African green monkey COS7, African green monkey Vero, human 293T, and human HeLa cells were cultured in Dulbecco’s modified Eagle’s medium supplemented with fetal bovine serum (8%) at 37°C with a 5% CO_2_ atmosphere. African green monkey Vero cell line was kindly provided by Dr. K. Morita (Okamoto et al., 2012). The other cell lines have been maintained in our laboratory.

### Expression Plasmids

The HIV-1 Gag-pol expression plasmid (pR8.91) was provided by Dr. D. Trono (Naldini et al., 1996). The VSV-G (pVSV-G) and LacZ-encoding HIV-1 vector gemone (pHIV-LacZ) expression plasmids were obtained from Dr. L. Chang through AIDS Reagent Program, NIAID, NIH, United States (Chang et al., 1999; Iwakuma et al., 1999). The expression plasmid of the X4-tropic HIV-1 HXB2 Env protein (pHXB2-Env) was provided by Yokomaku et al. (2004). The MLV Gag-pol expression plasmid was purchased from the TaKaRa, Japan. The C-terminally VSV-G epitope-tagged EZ-Wt and EZ-N expression plasmids were kindly provided by Dr. M. Arpin (Algrain et al., 1993). The EZ-TA and EZ-TD expression plasmids were constructed in our previous study (Kamiyama et al., 2018). African green monkey TRIM5α expression plasmid was obtained from Dr. T. Shioda (Nakayama et al., 2005, 2006). The VSV-G mutants (G124E and P127D) were kindly provided by Dr. J. Sakuragi (Ohishi et al., 2007).

### MLV Vector Expressing shRNA Against Ezrin

An MLV vector genome expression plasmid for shRNA expression (pSINsi-hH1) was purchased from TaKaRa. Two single strand DNAs were annealed and inserted into the pSINsi-hH1 plasmid. Their nucleotide sequences are 5’-GATCCGCCT GATTCTCGCGATTATTCTGTGTGCTGTCCAGAATAATCGC GAGAATCAGGCTTTTTTAT and CGATAAAAAAGCCTGAT TCTCGCGATTATTCTGGACAGCACACAGAATAATCGCGA GAATCAGGCG. The target sequence of the shEZ is identical to that of the siEZ used in this study. The shEZ-encoding MLV vector genome expression plasmid was transfected to 293T cells together with the MLV gag-pol and VSV-G expression plasmids. Culture media were changed to fresh media 1 day after the transfected to remove the transfection reagent, and cultured for additional 1 day. Culture supernatants from the transfected cells were added to COS7 cells, and the transduced COS7 cells were selected with G418 (Inalco Pharmaceuticals). G418-resistant cell pool was used in this experiment.

### Transduction Assay

COS7 cells (1 × 10^5^ cells) were seeded to a 6 cm-dish, and cultured for 2 days. The COS7 cells were transfected with the HIV-1 Gag-pol (1 μg), LacZ-encoding HIV-1 vector genome (1 μg), and VSV-G (1 μg) expression plasmids with the Fugene transfection reagent (Promega) (7 μL). Culture media of the transfected cells were changed to fresh media 24 h after the transfection, and cultured for 24 h. Culture media of the transfected cells were centrifuged to remove cells, and inoculated into HeLa cells. 2 days after the inoculation, the cells were stained with 5-bromo-4-chloro-3-indolyl-α-D-galactopyranoside (X-Gal) (Wako). Blue cells were counted to estimate transduction titers.

### Ezrin Silencing by siRNA

COS7 cells were transfected with the HIV-1 vector construction plasmids (1 μg each) together with siGFP or siEZ (200 pmol) in a 6-cm dish. Furthermore, COS7 cells were transfected with the HIV-1 vector construction plasmids and siEZ (200 pmol) together with pcDNA3.1 or EZ-Wt expression plasmid (1 μg) (about 0.2 pmol). This siEZ is identical to siRNA-E2 described previously (Kubo et al., 2008).

### Western Immunoblotting

COS7, Vero, or 293T cells were transfected as indicated or treated with concanamycin A (CMA) or MG-132 (Sigma-Aldrich). Cells were transfected with the HIV-1 vector construction plasmids. The transfected cells were cultured for 24 h, and washed with medium to remove the transfection reagent and plasmids. The cells were additionally cultured for 24 h, and cell lysates and culture supernatants were prepared from the cells. Cell lysates were subjected to SDS polyacrylamide gel electrophoresis (BioRad). Proteins were transferred to a PVDF membrane (Millipore). The membrane was treated with indicated first antibodies. As the first antibodies, rabbit anti-HIV-1 p24 (BioAcademia), goat anti-HIV-1 gp120 (Fitzgerald), goat anti-MLV p30 (ViroMed Biosafety Laboratories), mouse anti-actin (Santa Cruz), mouse anti-VSV-G (Sigma-Aldrich), and goat anti-ezrin (Santa Cruz) antibodies were used. When mouse antibodies were used as the first antibody, HRP-conjugated anti-mouse IgG (BioRad) was used. When the membrane was treated with a rabbit or goat antibody, HRP-conjugated protein G (BioRad) was used. Intensities of protein bands were measured by the ImageJ software.

### ELISA

Amount of HIV-1 p24 protein in cell lysates was quantitated by enzyme-linked immune servant assay (ZeptoMetrix).

### Cellular Localization

COS7 cells were transfected with the VSV-G expression plasmid together with the EZ-TA or EZ-TD expression plasmid in slide chambers. The transfected cells were permeabilized with methanol 2 days after the transfection, and treated with mouse anti-VSV-G and goat anti-ezrin antibodies and then with Cy3-conjugated anti-mouse IgG (Sigma-Aldrich) and FITC-conjugated anti-goat IgG antibodies. The cells were observed under a confocal fluorescent microscopy (Olympus).

### Statistics

Differences between two groups of data were determined using Student’s *t*-test. Statistical significance was set at *p* < 0.05 for all tests.

## Data Availability Statement

All datasets generated for this study are included in the article/Supplementary Material.

## Author Contributions

YK designed this study and wrote the manuscript. MI, KT, and YK performed the experiments in this study. MI, HH, TM, and YK analyzed the data.

## Conflict of Interest

The authors declare that the research was conducted in the absence of any commercial or financial relationships that could be construed as a potential conflict of interest.
