# Supplementary material for: Production of Vesicular Stomatitis Virus Glycoprotein-Pseudotyped Lentiviral Vector Is Enhanced by Ezrin Silencing
Source: Front Bioeng Biotechnol. 2020 Apr 29;8:368. doi: 10.3389/fbioe.2020.00368 (PMC7201057; doi:10.3389/fbioe.2020.00368)
Supplement: Supplementary file 1 [file Table_1.DOCX]

human MPKPINVRVT TMDAELEFAI QPNTTGKQLF DQVVKTIGLR EVWYFGLHYV 50

GM MPKPINVRVT TMDAELEFAI QPNTTGKQLF DQVVKTIGLR EVWYFGLQYV

human DNKGFPTWLK LDKKVSAQEV RKENPLQFKF RAKFYPEDVA EELIQDITQK 100

GM DNKGFPTWLK LDKKVSAQEV RKENPLQFKF RAKFFPEDVA EELIQDITQK

human LFFLQVKEGI LSDEIYCPPE TAVLLGSYAV QAKFGDYNKE VHKSGYLSSE 150

GM LFFLQVKEGI LSDEIYCPPE TAVLLGSYAV QAKFGDYNKE VHKSGYLSSE

human RLIPQRVMDQ HKLTRDQWED RIQVWHAEHR GMLKDNAMLE YLKIAQDLEM 200

GM RLIPQRVMDQ HKLTRDQWED RIQVWHAEHR GMLKDNAMLE YLKIAQDLEM

human YGINYFEIKN KKGTDLWLGV DALGLNIYEK DDKLTPKIGF PWSEIRNISF 250

GM YGINYFEIKN KKGTELWLGV DALGLNIYEK DDKLTPKIGF PWSEIRNISF

human NDKKFVIKPI DKKAPDFVFY APRLRINKRI LQLCMGNHEL YMRRRKPDTI 300

GM NDKKFVIKPI DKKAPDFVFY APRLRINKRI LQLCMGNHEL YMRRRKPDTI

human EVQQMKAQAR EEKHQKQLER QQLETEKKRR ETVEREKEQM MREKEELMLR 350

GM EVQQMKAQAR EEKHQKQLER QQLETEKKRR ETVEREKEQM MREKEELMLR

human LQDYEEKTKK AERELSEQIQ RALQLEEERK RAQEEAERLE ADRMAALRAK 400

GM LQDYEEKTKK AERELSEQIQ RALQLEEERK RAQEEAERLE ADRMAALRAK

human EELERQAVDQ IKSQEQLAAE LAEYTAKIAL LEEARRRKED EVEEWQHRAK 450

GM EELERQAVDQ IKSQEQLAAE LAEYTAKIAL LEEARRRKED EVEEWQHRAK

human EAQDDLVKTK EELHLVMTAP PPPPPPVYEP VSYHVQESLQ DEGAEPTGYS 500

GM EAQDDLVKTK EELHLVMTAP PPPPPPVYEP LSYHVQESLQ DEGTEPTGYS

human AELSSEGIRD DRNEEKRITE AEKNERVQRQ LLTLSSELSQ ARDENKRTHN 550

GM AELSSEGIRD DRNEEKRITE AEKNERVQRQ LLTLSSELSQ ARDENKRTHN

human DIIHNENMRQ GRDKYKTLRQ IRQGNTKQRI DEFEAL 586

GM DIIHNENMRQ GRDKYKTLRQ IRQGNTKQRI DEFEAL

**Supplementary figure 1** Amino acid sequences of human and green monkey (GM) ezrin. Black and red letters indicate identical and changed residues between human and green monkey ezrin.
